# The Link Between Alcoholism and Eating Disorders

**Published:** 1996

**Authors:** Lisa R. Lilenfeld, Walter H. Kaye

**Affiliations:** Lisa R. Lilenfeld, Ph.D., is a postdoctoral research fellow and Walter H. Kaye, M.D., is director of the Eating Disorders Module and professor of psychiatry at Western Psychiatric Institute and Clinic, University of Pittsburgh School of Medicine, Pittsburgh, Pennsylvania

**Keywords:** comorbidity, AODD (alcohol and other drug use disorder), eating disorder, female, bulimia nervosa, anorexia nervosa, epidemiology, etiology, treatment

## Abstract

The comorbidity between alcoholism and eating disorders, especially in young women, is well documented. Alcohol and other drug (AOD)-use disorders are particularly common in women with bulimia nervosa. Although the mechanisms underlying the coexistence of these disorders remain unknown, recent family epidemiology studies suggest that bulimia nervosa and AOD dependence are transmitted independently in families. Furthermore, bulimia nervosa generally develops before the onset of AOD dependence. Thus, factors other than addictive behavior may contribute to the development of bulimia nervosa in a substantial proportion of women. The comorbidity of AOD-use disorders with eating disorders has implications for the treatment of the affected patients.

Clinicians working with either alcoholic patients or patients with eating disorders have observed that both types of disorders frequently co-occur. Only recently, however, have researchers begun to investigate the reasons for this comorbidity. This article describes some characteristics of the most common eating disorders and reviews studies examining their comorbidity with alcoholism and other drug-use disorders.^1^ Moreover, the article presents the findings of preliminary analyses of the mechanisms that might contribute to the comorbidity of alcohol and other drug (AOD)-use disorders and eating disorders. Finally, the article reviews the implications of these findings for the treatment of patients suffering from both types of disorders.

## Common Eating Disorders

The two most common eating disorders are bulimia nervosa and anorexia nervosa. Both disorders primarily affect young women, with the usual ages of onset being between early and late adolescence for anorexia nervosa and between adolescence and early adulthood for bulimia nervosa. Only approximately 10 percent of all eating disorder cases occur in men. Because of this gender distribution, the vast majority of studies have investigated eating disorders only in women. Therefore, this review also focuses mainly on studies of women with eating disorders. Clinically diagnosable eating disorders are relatively rare in the general population. The lifetime prevalence rates are 1 to 3 percent for bulimia nervosa and 0.1 to 1 percent for anorexia nervosa (American Psychiatric Association [APA] 1994).

Bulimia nervosa is characterized by lack of control over food intake, leading to the recurrent consumption of large amounts of food in short periods of time (i.e., binge eating). These binge-eating episodes are interspersed with recurrent compensatory purging behavior(s), such as vomiting or laxative abuse, to prevent weight gain. In addition, the patients’ self-evaluations are unduly influenced by their body shape and weight (APA 1994).

Anorexia nervosa is characterized by the relentless pursuit of thinness, intense fears of becoming fat, and a distorted body image. People with anorexia nervosa experience substantial weight loss. In female patients, the resulting changes in the hormonal system also lead to the absence of menstruation (i.e., amenorrhea) (APA 1994). According to the APA’s *Diagnostic and Statistical Manual of Mental Disorders, Fourth Edition* (DSM–IV), two types of anorexia nervosa exist: a restricting type and a binge-eating/purging type. Patients with restricting anorexia nervosa lose weight primarily by extremely restricting their food intake. Patients with binge-eating/purging anorexia nervosa achieve or maintain a subnormal weight by regularly engaging in binge-eating and purging behavior in addition to restricting their food intake. Thus, binge-eating/ purging anorexia nervosa differs from bulimia nervosa in that the anorexic is severely underweight and amenorrheic, whereas the bulimic typically is of normal weight. These distinctions are important, because the rates of alcohol-use disorders vary among women with different eating disorders.

Another eating disorder described provisionally in the DSM–IV is binge-eating disorder (APA 1994). Like bulimia nervosa, it is characterized by the recurrent consumption of large amounts of food in short periods of time and lack of control during these binge-eating episodes. In contrast to bulimia nervosa, however, compensatory purging behavior does not occur. Only very limited research has been conducted on the rates of alcohol-use disorders among women with binge-eating disorder, and these studies have led to conflicting results (Spitzer et al. 1993; Wilson et al. 1993). Therefore, this review focuses only on the association of AOD-use disorders with bulimia nervosa and anorexia nervosa.

The causes and mechanisms (i.e., the etiology) underlying eating disorders remain unknown but most likely include genetic and environmental factors. Twin studies suggest that for bulimia nervosa, approximately 50 percent of the risk is attributable to genetic factors and 50 percent is attributable to the environment (Kendler et al. 1995). Moreover, the rates of eating disorders are elevated among the relatives of eating-disordered women (Lancelot et al. 1991), suggesting a familial etiology that may include both genetic and environmental effects.

## Rates of AOD-Use Disorders Among Women With Eating Disorders

Numerous studies have investigated the prevalence of AOD-use disorders among women with eating disorders. A recent review of 51 studies (Holderness et al. 1994) suggests that the rates of AOD-use disorders differ significantly among restricting anorexics, binge-eating/purging anorexics, and bulimics. Depending on the study analyzed, the rates of alcohol abuse or dependence among restricting anorexics ranged from 0 to 6 percent and the rates of other drug abuse or dependence (including amphetamines) ranged from 5 to 19 percent. In contrast, the corresponding rates in bulimics were significantly higher, ranging from 14 to 49 percent for alcohol abuse or dependence and from 8 to 36 percent for other drug abuse or dependence. Comparably high rates of alcohol-use disorders also were found in binge-eating/purging anorexics (see Laessle et al. 1989).

A recent survey of AOD-use disorders among women in the general population showed rates of 12 percent for alcohol abuse or dependence and 10 percent for other drug abuse or dependence (Kessler et al. 1994). Therefore, compared with women in the general population, women with bulimia nervosa or binge-eating/purging anorexia nervosa appear to have elevated rates of AOD-use disorders, whereas restricting anorexic women have lower rates of alcohol-use disorders but similar rates of other drug-use disorders. The relatively high rates of drug-use disorders, even among restricting anorexics, likely are attributable to the abuse of amphetamines and other drugs as a means of weight loss.

Women with different eating disorders also differ in their rates of regular alcohol use (i.e., not abuse or dependence): These rates were substantially higher among bulimics and binge-eating/purging anorexics (45 percent) than among restricting anorexics (11 percent) (Bulik et al. 1992). These variations could not be accounted for by age differences across groups. In addition, the rates of caffeine, laxative, and cigarette use were higher among bulimics and binge-eating/ purging anorexics than among restricting anorexics. Thus, bulimics and anorexics with bulimic symptoms are more likely to use and abuse AOD’s than are restricting anorexics.

## Rates of Eating Disorders Among Alcoholic Women

The frequency of eating disorders among alcoholic women has been studied less extensively than the frequency of alcohol-use disorders among eating-disordered women. Several studies have found, however, that the lifetime rates of any comorbid eating disorder among AOD-abusing women are significantly higher than in the general population, ranging from 15 to 32 percent (Higuchi et al. 1993; Beary et al. 1986; Hudson et al. 1992). With respect to specific eating disorders, both bulimia nervosa and binge-eating/purging anorexia nervosa are more common than restricting anorexia nervosa in both alcoholic populations (Higuchi et al. 1993; Beary et al. 1986) and mixed AOD-abusing populations (Hudson et al. 1992). According to these analyses, the rates of bulimia nervosa and binge-eating/purging anorexia nervosa range from 12 to 20 percent, whereas the rates of restricting anorexia nervosa range from 2 to 10 percent.

One study of eating disorders among female and male Japanese alcoholics (i.e., people with a diagnosis of alcohol abuse or dependence) in inpatient treatment found that 11 percent of the female alcoholics and 0.2 percent of the male alcoholics had lifetime histories of eating disorders (Higuchi et al. 1993). The very low rates of eating disorders among the male patients were consistent with lifetime prevalence rates of 0.01 to 0.1 percent for anorexia nervosa and 0.1 to 0.3 percent for bulimia nervosa observed among males in the general population. Thus, in contrast to female alcoholics, male alcoholics do not appear to experience significantly elevated rates of eating disorders compared with the general population.

A striking finding emerged when the researchers analyzed different age groups of female alcoholics: Seventy-two percent of all the female alcoholics under the age of 30 had lifetime histories of comorbid eating disorders, compared with 11 percent in the entire sample. Again, the majority of these patients (89 percent) suffered from either bulimia nervosa or binge-eating/ purging anorexia nervosa. Thus, the association between eating disorders with bulimic features and alcohol-use disorders appears to be particularly strong among young women. The reasons for this concentration of eating disorders among young women are unclear. Although bulimia nervosa has been defined only relatively recently (Russell 1979), its rates do not seem to be increasing (Frombonne 1996). Therefore, the age specificity most likely is not attributable to a cohort effect. Given the usual age of onset, however, younger women are more likely than older women to have current or recent histories of bulimia nervosa and therefore may be more reliable reporters of such histories.

Another study that examined the rates of eating disorders among female alcohol-dependent inpatients found that 30 percent of these women had lifetime histories of eating disorders (Beary et al. 1986). One-third of these women were diagnosed with anorexia nervosa, and two-thirds were diagnosed with bulimia nervosa. In both studies (Higuchi et al. 1993; Beary et al. 1986), the eating disorder preceded the onset of the alcohol-use disorder in the majority of cases.

In summary, the literature on both eating disorders and AOD-use disorders supports clinical observations of the frequent co-occurrence of both types of disorders. Furthermore, bulimia nervosa and the bulimic (i.e., binge-eating/purging) subtype of anorexia nervosa are much more commonly associated with alcohol-use disorders than restricting anorexia nervosa. These findings are consistent with past research that determined behavioral and personality trait differences among patients with different eating disorders. For example, binge-eating/purging anorexics and bulimics share certain traits characteristic of impulsive behavior and mood swings, whereas restricting anorexics consistently have been described as behaviorally restrained and compulsive (Vitousek and Manke 1994). Based on these characteristics, it is not surprising that bulimics and binge-eating/purging anorexics have high rates of AOD-use problems, whereas restricting anorexics do not exhibit AOD-use problems or other impulsive behaviors.

## Patterns of AOD-Use and Eating Disorders in Families

Although studies have confirmed that eating disorders and AOD-use disorders frequently coexist, the mechanisms underlying this comorbidity have not been elucidated. At least four potential explanations exist for the high rates of co-morbidity (Klein and Riso 1993):

- Both disorders are different manifestations of a shared underlying etiology.
- The two disorders have different causes, but the presence of one disorder may increase a person’s chances of developing the other.
- An independent disorder causes both disorders.
- The two disorders have some risk factors in common, whereas other risk factors are specific to each disorder.

The first hypothesis of a common etiology has been studied most extensively. Supporters of this hypothesis believe that both eating disorders and AOD-use disorders may be manifestations of an underlying predisposition toward impulsivity or may result from a common mechanism involving endogenous opioids (Wilson 1991). Endogenous opioids—compounds that occur naturally in the body and act like opiates—have been shown to play a role in regulating alcohol consumption as well as appetite (Swift 1995; Jackson et al. 1992). One way to investigate this hypothesis is to use a family study design, in which the prevalence of a disorder (e.g., alcoholism) is compared between the relatives of a particular patient population (e.g., patients with eating disorders) and the relatives of the normal control subjects. Family studies are a well-accepted approach to studying the mechanisms underlying the frequent coexistence of two disorders (see Merikangas et al. 1994).

Very few studies have investigated the possible roles of transmissible genetic or environmental familial factors in the comorbidity of eating disorders and alcohol-use disorders. In a recent family study, Kaye and colleagues (in press) assessed the presence of psychiatric disorders, including AOD-use disorders, among restricting anorexics, bulimics, and non-eating-disordered women and their first-degree relatives using direct structured interviews. One question addressed by this study was whether bulimia nervosa and AOD-use disorders represent alternative observable manifestations (i.e., phenotypic expressions) of a shared transmissible factor. If so, family members of bulimics should exhibit elevated lifetime rates of AOD dependence, regardless of the presence or absence of AOD dependence in the bulimics themselves. Alternatively, bulimia nervosa and AOD dependence could be coexisting but separate disorders (Merikangas et al. 1994). In that case, only relatives of bulimic subjects with coexisting AOD dependence would have a higher prevalence of AOD dependence, whereas the relatives of bulimic subjects without coexisting AOD dependence would have AOD dependence rates similar to those among relatives of nonbulimic women.

Accordingly, the researchers divided the bulimic subjects into two groups: those with and those without lifetime histories of comorbid AOD dependence. When the rates of AOD dependence were examined in the first-degree relatives of both groups, the prevalence was elevated only among the relatives of bulimics with comorbid AOD dependence but not among the relatives of non-AOD-dependent bulimics. The analyses produced similar, though less robust, results when the rates of not only AOD dependence but also AOD abuse and dependence were evaluated. These findings refute the hypothesis that eating disorders and AOD-use disorders are different manifestations of a shared underlying etiology and indicate instead that both disorders are attributable to independent transmissible factors.

If many bulimic women do *not* exhibit familial vulnerability to AOD-use problems, factors other than addictions may contribute to the development of bulimia nervosa in a substantial proportion of women. These findings raise the possibility that two bulimic subtypes exist. The first type would represent a “multi-impulsive” subtype, with a familial and personal history of AOD-use problems. The second type may best be described as an “anorexic-like” subtype, with personality and behavioral traits similar to restricting anorexics (e.g., behavioral restraint and compulsiveness). Similar to restricting anorexics, these bulimics have no extensive personal or family histories of AOD-use problems.

Other recently published studies also suggest that alcoholism and bulimia nervosa do not share a common etiology. For example, Schuckit and colleagues (1996) conducted structured interviews with 2,283 women and 1,982 men as part of the Collaborative Study on the Genetics of Alcoholism. The sample consisted of women and men who were alcohol dependent^2^ as well as their first-and second-degree relatives. The subjects included both “primary alcoholics,” whose onset of alcohol dependence preceded the onset of any other psychiatric disorder, and “secondary alcoholics,” whose onset of alcohol dependence followed the onset of one or more comorbid disorders. The study did not detect significantly higher rates of eating disorders among the relatives of primary alcoholics or among the relatives of primary and secondary alcoholics combined than among the relatives of nonalcoholic comparison subjects. The authors concluded that “any relationship that might exist between anorexia nervosa or bulimia nervosa, on the one hand, and alcohol dependence, on the other, is not likely to represent a strong genetic linkage with alcohol dependence itself” (Schuckit et al. 1996, p. 80).

Genetic analyses of six major psychiatric disorders in women, including bulimia nervosa and alcoholism, supported the existence of separate genetic liabilities for eating disorders and alcoholism (Kendler et al. 1995). The study included 1,030 female twin pairs, ascertained from the general population through the Virginia Twin Registry, who were evaluated through direct interviews. Statistical analyses examining correlations of multiple variables between and across twin pairs indicated that bulimia nervosa and alcoholism were best explained by two different genetic factors. In fact, most genetic factors that influenced the women’s vulnerability to alcoholism appeared to be distinct from the genetic factors determining the risks for other disorders, including bulimia nervosa.

Thus, the three epidemiological studies described here suggest that although bulimia nervosa and alcoholism frequently coexist, they apparently do not share an underlying familial or genetic liability. Instead, the two disorders are likely to result from independent causal factors.

The other three potential explanations for the high rates of comorbidity between alcoholism and eating disorders have been examined less thoroughly. Most studies investigating the hypothesis that the presence of one disorder may increase the chances of developing the other disorder found that the onset of bulimia nervosa generally preceded the onset of alcohol dependence (see Higuchi et al. 1993). Although this observation is not surprising, given the different ages of onset for the two disorders, it does not resolve the question of whether this temporal pattern also indicates that bulimia nervosa somehow causes the onset of alcohol dependence.

The theory that an independent disorder can cause both eating disorders and alcoholism also has not been well studied. One potentially fruitful line of research would be to examine the relationship among anxiety disorders, bulimia nervosa, and alcoholism, because anxiety disorders frequently co-occur with both of the other disorders (Brewerton et al. 1995; Brady and Lydiard 1993).

The most likely—although also not well-investigated—explanation of the frequent comorbidity of eating disorders, particularly bulimia nervosa, and AOD-use disorders may be that people with both types of disorders share some underlying traits, such as periodic behavior disinhibition and difficulty modulating feelings or emotions (i.e., affect). In addition to these shared traits, other etiologic factors likely exist that are specific to each disorder. Clearly, more research is needed to further evaluate this hypothesis.

## Treatment Implications

Over the past decade, several researchers have hypothesized that bulimia nervosa, like alcoholism, is a type of addictive disorder (Vandereycken 1990; Wilson 1991). Both alcoholics and bulimics describe feelings of “craving” and a “loss of control” over a substance (i.e., alcohol or food), become preoccupied with the substance, and repeatedly attempt to stop their pattern of overconsumption. Moreover, both disorders can impair a person’s physical and social functioning and may involve deception and secrecy.

The addiction model of eating disorders (Wilson 1991) has contributed to the notion that eating disorders and AOD-use disorders may respond to similar treatment approaches. In fact, many bulimics are treated in 12-step-like programs, and Johnson and Sansone (1993) describe a program in which more traditional therapy modalities are combined with a 12-step component. To date, however, no rigorous, scientifically designed studies have demonstrated the benefits of a 12-step approach for treating bulimia nervosa. Moreover, because the 3 recent epidemiological studies have demonstrated that bulimia nervosa and AOD-use disorders have independent liabilities, it is possible that 12-step-like programs may not be useful for bulimics without coexisting AOD-use problems.

In contrast, other therapies, such as cognitive-behavioral therapy (CBT) and antidepressant medication, have proven useful in treating eating disorders (Abbott and Mitchell 1993). CBT focuses on identifying and restructuring distorted thoughts (e.g., a negative body image and fears of fatness), which in turn influence behavior. Other, more specific behavioral strategies used in treating eating disorders may include imposing a time-delay between a binge episode and the vomiting episode that usually immediately follows binge eating, with the goal to gradually increase the time delay.

The most effective treatment approaches for alcoholism and eating disorders also will likely differ, because different behaviors must be addressed for both types of disorders. The primary focus in alcoholism treatment is to avoid consuming the substance, whereas the focus in bulimia nervosa treatment is to change the manner in which the substance (i.e., food) is consumed. Many bulimics and binge-eating/purging anorexics have difficulty modulating food intake and often alternate between periods of imposed food restriction and overconsumption combined with purging behaviors. Treatment must take into consideration both these consumption patterns as well as a disturbed body image. Accordingly, cognitive-behavioral approaches addressing all these issues differ greatly from the 12-step programs commonly used to treat alcoholics.

Few empirical data exist to help identify and treat patients with coexisting eating disorders and AOD-use disorders. Still, the frequent comorbidity of these disorders underscores the necessity of determining whether eating disorders are present, especially among young, AOD-abusing women entering treatment. Adequate assessment is even more important, because alcoholic, eating-disordered patients also are likely to engage in other behaviors common among “multi-impulsive bulimics,” such as shoplifting, suicide attempts, or self-mutilating behavior (Suzuki et al. 1994). These characteristics may have significant implications for the clinical management of such patients.

AOD-abusing patients with coexisting eating disorders should receive thorough medical assessments and nutritional consultations. The management of these patients should include monitoring their weight, food intake, and purging behavior as well as assessing their cardiac, fluid, and mineral (i.e., electrolyte) statuses. The patients should be observed during and after each meal, with supervised bathroom use to minimize purging opportunities. Although monitoring eating-disordered patients in an AOD-abuse treatment facility may be challenging and labor intensive, it is necessary for treatment.

Patients with comorbid AOD-use and eating disorders can pose a particular challenge for the individual clinician or the staff of a treatment facility. These patients may represent a group distinct from patients who only have an AOD-use disorder or an eating disorder. They may require different, more varied, and more intensive assessment and treatment approaches. Researchers and clinicians still have not identified the most beneficial treatment approaches for these patients, and future treatment outcome studies must address this difficult patient population.

## Footnotes

1 Many people with eating disorders abuse not only alcohol but also other drugs (e.g., amphetamines), and the studies mentioned in this article frequently discuss alcohol and other drug use in general.

2 Alcohol dependence in this study was defined according to the criteria of the American Psychiatric Association’s *Diagnostic and Statistical Manual of Mental Disorders, Third Edition, Revised*.
